# Solar Heat Flux Suppression on Optical Antenna of Geosynchronous Earth Orbit Satellite-Borne Lasercom Sensor

**DOI:** 10.3390/s24155005

**Published:** 2024-08-02

**Authors:** Ming Liu, Hongwei Zhao, Chengwei Zhu, Guanyu Wen

**Affiliations:** 1 School of Mechanical and Aerospace Engineering, Jilin University, Changchun 130025, China; hwzhao@jlu.edu.cn; 2 Changchun Observatory, National Astronomical Observatories, CAS, Changchun 130117, China; zhucw@cho.ac.cn (C.Z.); wengy@cho.ac.cn (G.W.)

**Keywords:** GEO satellite-borne lasercom sensor, optical antenna, solar heat flux, solar avoidance strategy, thermal control solution

## Abstract

The objective of this article is to examine potential techniques for suppressing solar heat flow on the optical antenna of a laser communication sensor. Firstly, the characteristics of the geosynchronous Earth orbit’s (GEO) space radiation environment are analysed, and a combined passive and active thermal control solution is proposed. Secondly, the temperature distribution of the lasercom sensor under extreme operating conditions is simulated utilising IDEAS-TMG (6.8 NX Series) software, which employs Monte Carlo and radiative heat transfer numerical calculation methods. Finally, a strategy for avoiding direct sunlight around midnight is proposed. The simulation results demonstrated that the thermal control solution and solar avoidance strategy proposed in this paper achieved long-term fine-stable control of the temperature field of the optical antenna, which met the thermal permissible communication hours per daily orbit cycle in excess of 14 h per day.

## 1. Introduction

Lasercom technology has advantages, such as a high data transmission rate, low power consumption, and strong anti-interference capability, etc. [1]. It provides a link bandwidth of tens of Gbps, much higher than that of a traditional microwave communication system, making it an effective means to achieve high-speed communication, and it represents the development direction of the new generation of high-speed and real-time communication satellites [2]. Many countries have been successful in the experimental verification and application of space laser communication between low Earth orbit (LEO) satellites and geostationary Earth orbit (GEO) satellites, as well as LEO–LEO, LEO–ground, and lunar platform–ground communication [3,4,5,6]. The object of this research is a lasercom sensor onboard a GEO satellite, which is exposed to solar radiation, resulting in the degradation of communication quality or even permanent failure, seriously affecting the sensor’s lifespan and the thermal permissible communication duration [7]. A variety of strategies have been explored to alleviate the impact of solar heat flux. These strategies encompass passive thermal control techniques, active thermal management systems, and the development of advanced materials. Researchers have explored passive thermal management strategies that do not require external power or active control [8]. One such approach is the use of highly reflective and low-emissivity coatings on the optical antenna surfaces [9,10,11]. These coatings can significantly reduce the solar absorption and re-radiation of thermal energy, effectively mitigating the impact of solar heat flux [12,13,14]. Another passive technique is the incorporation of thermal insulation materials, such as multi-layer insulation (MLI) or aerogel-based insulation, within the antenna structure. These materials act as thermal barriers, minimising heat transfer from the external environment to the critical optical components [15]. Active thermal control strategies involve the use of powered systems to actively regulate the temperature of the optical antenna [16,17,18,19]. One example is the implementation of thermoelectric coolers (TECs) or cryogenic coolers to keep the optical components at the desired operating temperature [20,21]. These active cooling systems can effectively counteract the solar heat flux and maintain the required thermal stability. The development of advanced materials is crucial for effective thermal management. Recent advances in nanomaterials and metamaterials have opened new avenues for thermal regulation [22,23,24]. As documented in the aforementioned literature, despite the considerable advancements in solar heat flux suppression strategies for optical antennas, several challenges remain. One of the most significant challenges is the integration of thermal control solutions with existing satellite architectures. Many proposed solutions, particularly active thermal management systems, may necessitate substantial modifications to current satellite designs, which could result in increased complexity and costs. Secondly, the development of integrated thermal solutions that effectively combine passive and active techniques represents a promising avenue. Optimising the synergies between these two solutions while addressing the challenges of system complexity and power consumption is crucial to achieving comprehensive and reliable thermal control for GEO satellite-borne lasercom optical antennas.

This paper presents a study on the solar heat flux suppression of a satellite-borne lasercom sensor. Firstly, the characteristics of the GEO’s complex space radiation environment are analysed, and a combined passive and active thermal control solution is proposed based on the temperature distribution of the sensor caused by solar heat flux. The integrated thermal control solution is a modular thermal management system that can readily be adapted to various satellite designs. The integrated thermal control solution employs heating sheets and thermoelectric coolers to actively regulate the temperature of the sensor, while passive thermal control explores the optimisation of the baffle’s geometry and the combination of annular heat pipe rings, flexible heat conductors, and a radiating plate coated with a special film to evenly divert the heat radiation, thus reducing the heat load. Secondly, thermal simulations are then conducted to analyse the temperature of the optical components under two extreme operating conditions to evaluate the heat dissipation capability of the thermal control solutions. Finally, a strategy for avoiding direct sunlight around midnight is proposed, which cooperates with the combined passive and active thermal control solution to achieve integrated thermal management system optimisation. This results in thermal control power consumption of only 30 W for the optical antenna, which allows the temperature of the primary and secondary reflectors to be maintained within the permissible range, thus ensuring the stable and normal operation of the lasercom sensor in orbit. Furthermore, the thermal permitted hours of satellite–Earth laser communication per daily orbit cycle are finally determined.

## 2. GEO Satellite-Borne Lasercom Sensor

**Figure 1:**
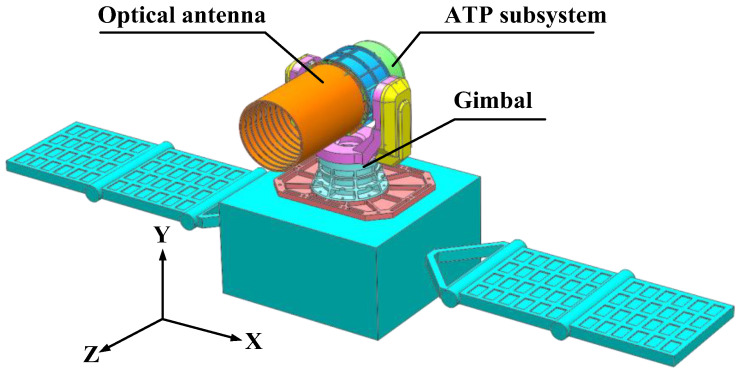
A schematic of the satellite-borne lasercom sensor’s composition.

The object of this research is a lasercom sensor on board a GEO satellite, which primarily establishes a communication link with the Optical Ground Station (OGS). The lasercom sensor is mounted on the +Y side of the satellite platform and consists of an optical antenna, an ATP (Acquisition, Tracking, Pointing) subsystem, and a gimbal, as shown in Figure 1. The optical axis orientation (+Z) of the lasercom sensor always points towards the OGS and rotates synchronously with the Earth. The instantaneous coordinates of the lasercom sensor are defined as follows, and will be frequently employed in subsequent analyses: the +Z direction represents the side of the satellite directed towards the ground, the ±X direction denotes the direction of the satellite travelling around the Earth in the GEO, i.e., the east–west direction, and the ±Y direction is determined by the right-hand rule, i.e., the north–south direction.

**Figure 2:**
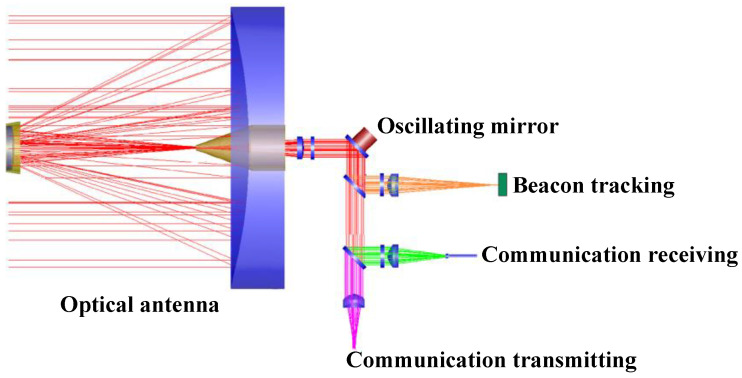
An optical schematic of the lasercom sensor.

The lasercom sensor is functionally divided into four units: communication transmitting, communication receiving, beacon tracking, and servo control. The units of communication transmitting, communication receiving, and beacon tracking share a single Cassegrain antenna and a single oscillating mirror. The optical antenna compresses the light beam to collect energy and shoots out parallel light, which is then collected by the APT subsystem. The beam splitter and filter are employed to facilitate the detection of the beacon and the separation of the communication-receiving and -transmitting units. An optical schematic of the lasercom sensor is shown in Figure 2.

**Figure 3:**
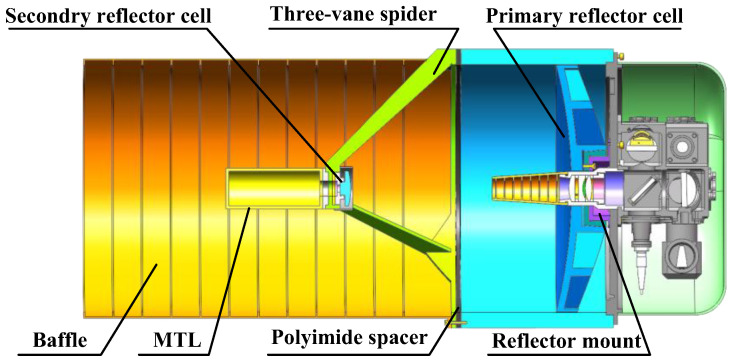
A schematic of the optical antenna’s composition.

In the GEO–ground laser link, the link distance ranges from 36,935 km to 39,471 km, and the extremely narrow divergence angle of the beam requires a high-gain optical antenna for long-distance communication. The optical antenna described in this paper employs a Cassegrain configuration, in which the primary mirror is a parabolic reflector, and the secondary mirror is a hyperbolic reflector. The optical antenna comprises a primary reflector cell, a telescope tube, a secondary reflector cell, a three-vane spider, and a baffle, as shown in Figure 3.

The primary reflector is mounted on a central hub in the primary reflector cell, and the secondary reflector is held by an A-shaped three-vane spider at the top of the telescope tube. The primary and secondary reflector cells, as well as the baffle, are connected by the telescope tube, with the baffle thermally mounted to the telescope tube by a polyimide spacer. The material properties of the optical antenna components are presented in Table 1.

The Cassegrain antenna is used to collect energy, restrict beam spread, block ambient light, and suppress solar heat flux, thereby enabling the optical antenna to achieve high laser power gain. The performance requirements of the optical antenna and the related parameters are presented in Table 2.

The objective of the solar heat flux suppression solutions explored in this paper is to ensure that the primary and secondary reflectors of the optical antenna are kept within specified temperature ranges in two modes, as presented in Table 2. The excitation load for the suppression solutions is the solar heat flux, to which the lasercom sensor is subjected during GEO sensor operation. Furthermore, these solutions are contingent upon two boundary conditions: firstly, the thermal control power of the optical antenna must not exceed 50 W; secondly, the lasercom sensor mount’s surface temperature is set between −10 °C and 10 °C.

It is important to recognise that the duration of lasercom availability is directly affected by the wavefront quality of the optical antenna, atmospheric absorption and scattering, and cloud cover [25,26,27]. This is a very complex subject. However, the primary focus of this paper and Table 2 is the thermal permissible laser communication hours per daily orbit cycle. It is essential to keep the temperature of the optical antenna’s primary and secondary reflectors of the optical antenna within the permissible range to guarantee optimal wavefront quality and prevent irreversible damage to the reflector film layer, thus providing high-quality optics for laser communication.

## 3. Space Radiation Analysis of the Lasercom Sensor in GEO

The lasercom sensor relays data from the GEO to the OGS with its optical axis pointing towards the ground. The field of view of the OGS to the optical antenna is approximately 17.4°, which is considerably larger than the acquisition field of view of the lasercom sensor (4 mrad). Consequently, when communicating with the ground station, the satellite-borne lasercom sensor’s field of view is filled with the Earth’s background and sunlight, without the presence of other point light sources, such as stars.

Firstly, the irradiance of the Earth’s background radiation is calculated as follows [28]:

(1) $$\left\{\begin{array}{c} E\left(\lambda \right)=E_{e}\left(\lambda \right)+E_{s}\left(\lambda \right)\\ E_{e}\left(\lambda \right)=\frac{c_{1}}{\lambda^{5}\left(e^{\frac{c_{2}}{\lambda T_{e}}}-1\right)}\left(\frac{r}{r+h}\right)\\ E_{s}\left(\lambda \right)=\frac{c_{1}}{\lambda^{5}\left(e^{\frac{c_{2}}{\lambda T_{s}}}-1\right)}R_{e}\left(\lambda \right)\left(\frac{r_{s}}{1AU}\right) \end{array}\right.,$$

In this expression, *E_e_*(*λ*) represents the spectral irradiation emitted by the Earth, while *E_s_*(*λ*) denotes the reflected solar spectral radiation from the Earth. The first radiation constant, *C*_1_, is equal to 3.742 × 10^−16^ W·m^2^, while the second radiation constant, *C*_2_, is equal to 1.439 × 10^4^ μm·K, and *λ* is the wavelength of the laser. The blackbody equivalent temperature of the Earth, *T_e_*, is 293 K. The Earth’s radius, *r*, is 6371 km, while the GEO altitude, *h*, is 35,786 km. The blackbody equivalent temperature of the Sun, *T_s_*, is 5762 K. The solar altitude, 1*AU*, is 1.5 × 10^8^ km. *R_e_*(*λ*) represents the Earth’s spectral reflectance of the Sun.

The Earth’s background radiation energy, which can eventually enter the optical antenna, is calculated as follows:

(2) $$\varphi_{D}=E\left(\lambda \right)S_{0}\sin\left(\frac{\omega }{2}\right)\lambda ,$$

where the acquisition field of view of the lasercom sensor, *ω*, is equal to 4 mrad, and the incident pupil area, *S*_0_, is equal to 0.07 m^2^. The power of the Earth’s background radiation entering the sensor at the wavelengths of the communication light (800 nm) and the beacon light (1500 nm) is 2.7 × 10^−11^ W and 0.96 × 10^−12^ W, respectively, which is much less than the optical power of the received beacon light and communication light (0.54 × 10^−9^ W and 2.7 × 10^−7^ W, respectively). Consequently, the satellite-borne lasercom sensor is only slightly affected by the Earth’s background radiation, and is mainly affected by radiation from the Sun.

**Figure 4:**
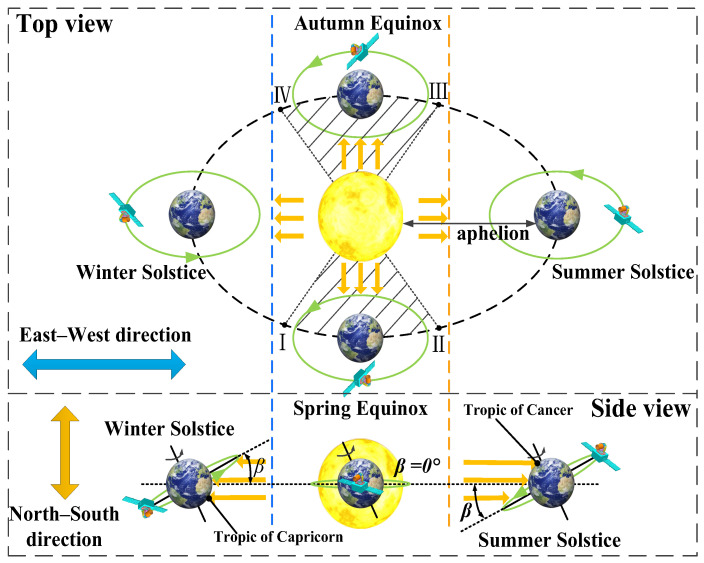
The relative positions of the satellite, Sun, and Earth over the course of a year.

**Figure 5:**
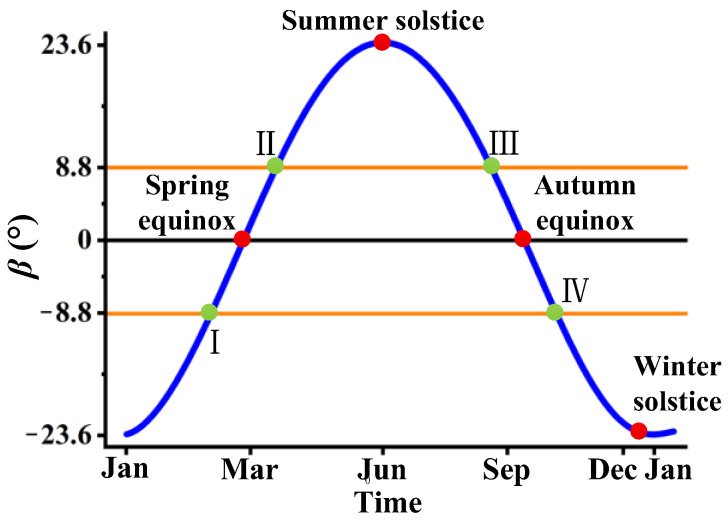
The variation in the angle (*β*) of sunlight in the north–south direction relative to the Earth’s equatorial plane with the seasons.

The main factors affecting the amount of solar heat flux reaching the lasercom sensor are as follows: the relative position of the satellite, Sun, and Earth, the Earth’s revolution and seasonal variations, the Earth’s rotation, and the attitude of the lasercom sensor. The relative positions of the satellite, Sun, and Earth over the course of a year are shown in Figure 4. Figure 5 shows the variation in the angle (*β*) of sunlight in the north–south direction relative to the Earth’s equatorial plane with the seasons.

Figure 4 illustrates that as the Earth revolves around the Sun, it reaches aphelion, where the Sun is directed towards the Tropic of Cancer during the summer solstice, and perihelion, where the Sun is directed towards the Tropic of Capricorn during the winter solstice. The Sun’s light moves back and forth from east to west in a 24 h cycle as the Earth rotates, and the angle between the sunlight and the Earth’s equatorial plane in the north–south direction varies between −23°26′ and 23°26′ in a yearly cycle, in accordance with the Earth’s rotation around the Sun. Figure 4 illustrates the phenomenon of the satellite entering the Earth’s shadow area during the spring and autumn equinoxes, with the shadow area delineated by the points labelled I~II and III~IV in Figure 4. This phenomenon occurs dozens of days before and after the spring and autumn equinoxes, with the satellite entering the Earth’s shadow at approximately midnight. This phenomenon is referred to as ‘midnight Sun disappearance’. During this period, the Sun, Earth, and satellite are in alignment, resulting in the camera being shielded from solar radiation and heat flow. During the remainder of the year, the satellite is exposed to the Sun throughout the day. Figure 5 illustrates that the angle (*β*) of sunlight in the north–south direction relative to the Earth’s equatorial plane reaches a maximum value of +23°26′ during the summer solstice and −23°26′ during the winter solstice. The critical angle for satellites to enter and leave the Earth’s shadow zone is ±8.8° (corresponding to points I, II, III, and IV in Figure 4); that is to say, the ‘midnight Sun disappearance’ phenomenon occurs if *β* ≤ |±8.8°| and, conversely, when *β* ≥ |±8.8°|, the satellite-borne lasercom sensor will be exposed to the Sun throughout the entire day. At the spring or autumn equinox, *β* = 0°, the ‘midnight Sun disappearance’ phenomenon may last up to 72 min. Thereafter, the duration for which the satellite remains in the Earth’s shadow zone increases as |*β*| decreases.

**Figure 6:**
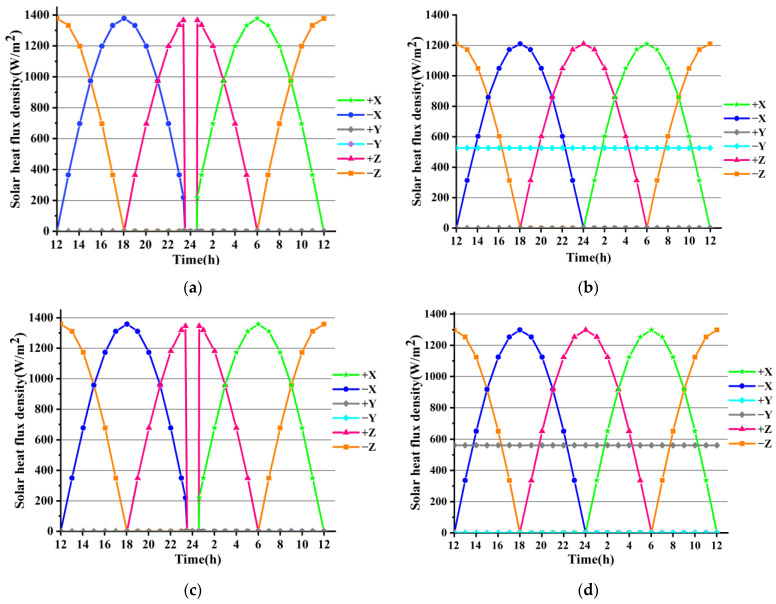
The solar heat flux density over the six surfaces of the satellite-borne lasercom sensor. Solar heat flux density during (**a**) spring equinox, (**b**) summer solstice, (**c**) autumn equinox, and (**d**) winter solstice.

IDEAS (6.8 NX Series) software was employed to analyse the solar heat flux density over the six surfaces of the satellite-borne lasercom sensor as the seasons changed, as shown in Figure 6.

Figure 6 reveals the following: (1) The solar heat flux density is greatest during the vernal equinox, with a value of approximately 1345 W/m^2^. (2) The solar heat flux on the +/−X side of the lasercom sensor alternates throughout each day (24 h); for the first 12 h, it is exposed to the Sun, and for the remaining 12 h, it is completely shielded from solar radiation. (3) When the solar heat flux on the +/−X side decreases, the solar heat flux on the +Z side (optical entrance) increases. (4) The +Y side is exposed to solar radiation during the winter solstice, while the −Y side is in the leeward zone, and the opposite is true during the summer solstice. Furthermore, during both the equinoxes, the +/−Y side is shielded from the solar heat flux. (5) The ‘midnight Sun disappearance’ phenomenon occurs around midnight during the vernal/autumnal equinox, when the lasercom sensor shields in the Earth’s rear shadow region for a maximum of approximately 72 min.

## 4. Passive and Active Thermal Control Design

Sunlight shining into the optical antenna will result in an increase in the temperature of the primary and secondary reflectors. Once the temperature exceeds the temperature range index in Table 1, the wavefront aberration of the optical antenna will increase, leading to a deterioration in communication quality [29]. This can be mitigated by optimising the design of the baffle and by implementing active and passive thermal control measures. The baffle can block most of the sunlight of large inclination, and passive and active thermal control enables the temperature of the reflectors to be kept within the temperature index with the lowest power consumption. However, as soon as the sunlight reaches the optical antenna and exceeds the thermal control capacity, until the temperature control index is exceeded, the lasercom sensor can only interrupt communication and rotate its view to avoid the solar radiation

### 4.1. Baffle Design

**Figure 7:**
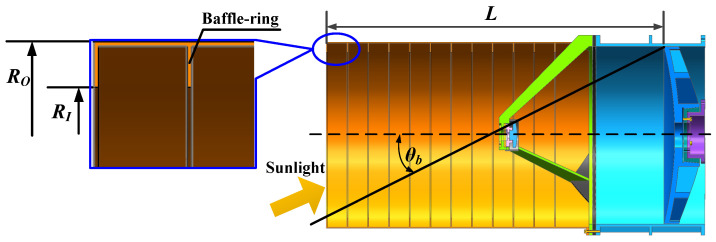
The structural parameters of the baffle and thermal balance.

The angle at which the sunlight hits the primary reflector through the baffle is defined as the ‘solar-blocking angle’ (*θ_b_*). When the incidence angle is less than this ‘solar-blocking angle’, the thermal control system begins to operate, sometimes even requiring the interruption of communication and rotation of the optical axis to avoid direct sunlight. The ‘solar-blocking angle’ (*θ_b_*) is directly related to the shape and size of the baffle, so the baffle should be designed to reduce the interruption of communication and avoid direct sunlight, thus extending the thermal permissible lasercom duration. The structural parameters of the baffle are shown in Figure 7.

The above conclusion, as illustrated in Figure 5, indicates that the angle between the sunlight and the Earth’s equatorial plane in the north–south direction varies between −23°26′ and 23°26′ in a yearly cycle. Consequently, the ‘solar-blocking angle’ (*θ_b_*) can be set to 23°, and the length of the baffle can be calculated as follows:

(3) $$L=\frac{D}{tan\theta_{b}},$$

where *D* is the diameter of the primary reflector and *L* is the length of the baffle (*L* = 600 mm).

**Figure 8:**
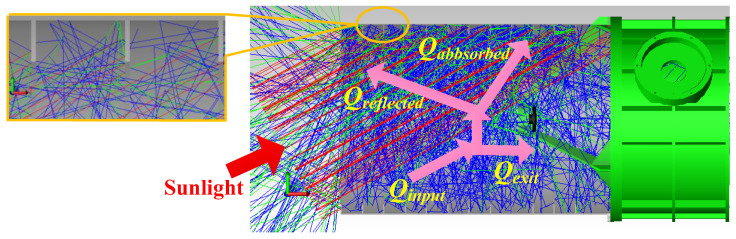
Solar beam tracing into the optical antenna.

To determine the number of baffle-rings and the height of *R_O_* in Figure 7, TRACEPRO 7.0 software was adopted to simulate the solar beam tracing into the optical antenna at the ‘solar-blocking angle’, as shown in Figure 8, where *Q_input_* represents the angularly integrated incident heat energy, which is the total incident energy over the aperture diameter, defined by a radius of *R_O_*, and *Q_reflect_* represents the fraction heat energy absorbed by the baffle.

**Figure 9:**
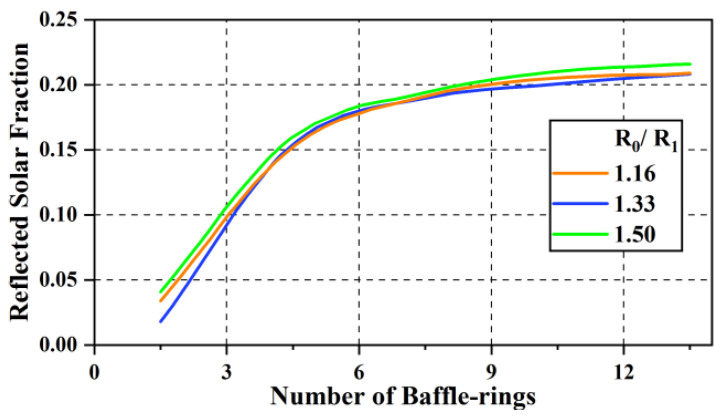
Solar-reflecting efficiency of the baffle.

With this beam-tracing simulation, the number of baffle-rings and the height of *R_O_* can be determined from the reflection efficiency of the baffle, as shown in Figure 9, where the reflected solar fraction (*F_r_*) is *Q_reflect_*/(*Q_input_* − *Q_output_*).

Observing Figure 9, it is easy to see that the solar-reflecting efficiency is highest when the number of baffle-rings is ≥12 and *R_O_*/*R_I_* = 1.5. However, *R_O_*/*R_I_* makes the aperture of the baffle too large, resulting in a low base frequency, and is also limited by the envelope dimensions of the rocket launch fairing, so the number of baffle-rings is set to 12 and *R_O_*/*R_I_* is set to 1.16.

### 4.2. A Combined Passive and Active Thermal Control Solution

**Figure 10:**
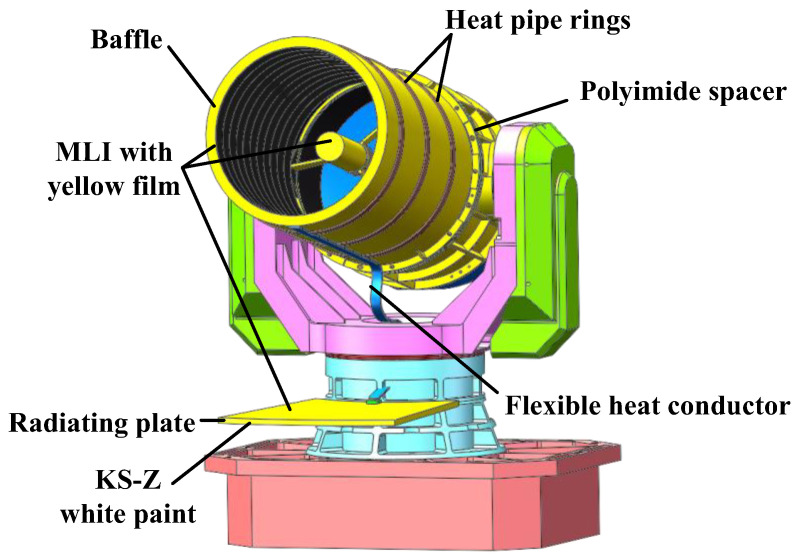
The thermal control measures and layout of the satellite-borne lasercom sensor.

According to the performance requirements and thermal control indexes in Table 2 and the solar heat flux input conditions in Figure 6, a combined passive–active thermal control solution was adopted, with passive thermal control as the primary mode. Passive thermal control measures include the use of Multi-Layer Insulation (MLI), polyimide spacers, and thermal paint or film, and the deployment of thermal conductors and radiating plates to dissipate heat. The active thermal control measures include a closed-loop temperature control system consisting of heating sheets, thermoelectric coolers (TECs), and temperature sensors. The thermal control measures and layout of the satellite-borne lasercom sensor are shown in Figure 10.

The solar heat flux characteristics, as shown in Figure 6, indicate that both the ±X and ±Z surfaces are unsuitable for use as radiating plates, given that the solar heat flux on both the +/−X and +/−Z sides alternates throughout each day (24 h). During the summer and winter solstices, only one side of the Y-direction is exposed to sunlight, while the external solar heat flux on the other side is zero. Furthermore, both the +Y and −Y sides are shielded from the solar heat flux during both equinoxes. Therefore, the +Y and −Y sides are suitable for use as radiating plates. The Multi-Layer Insulation (MLI) is wrapped over the +Y side of the radiating plate, and then, the yellow film, with a solar absorptivity (*α_s_*) of 0.36, is wrapped over the outermost layer of the MLI. The +Y side is employed as a heat-isolating surface, with the MLI and yellow film reducing radiant heat transfer and serving as a heat shield. The −Y side is employed as a heat-expelling surface and is coated with KS-Z white paint with a solar absorptivity (*α_s_*) of 0.13.

The baffle shown in Figure 10 is constructed from composite carbon fibre and is attached to the telescope tube by a polyimide spacer. Three groups of uniform temperature heat pipe rings, constructed from copper, are arranged in the circumferential direction of the baffle, which can quickly diffuse the heat in all directions, while flexible heat conductors are arranged in the axial direction to conduct the heat to the radiating plate. Ten units of MLI are wrapped around the baffle, and then, a yellow film with a solar absorptivity (*α_s_*) of 0.36 is wrapped around the outermost layer of the MLI. Four heating zones are set up for closed-loop temperature control on the +X, −X, +Y, and −Y sides of the baffle cavity.

**Figure 11:**
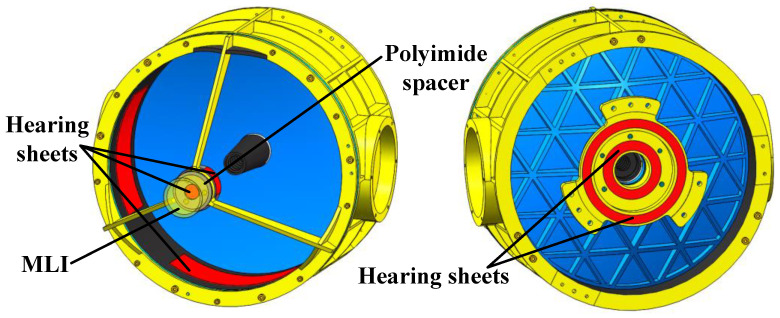
The thermal control solution inside the optical antenna.

Four heating sheets are affixed in the circumferential direction of the secondary reflector baffle and to the posterior (Z-axis) of the secondary reflector mount, and then, 20 units of MLI are wrapped around the outermost layer, as shown in Figure 11. Five heating zones are set up on the +X, −X, +Y, and −Y sides of the primary reflector cell, as well as on the −Z side of the primary reflector mount. The thermal control solution inside the optical antenna is shown in Figure 11, and the power consumption of the active thermal management system for optical antenna is presented in Table 3.

### 4.3. Thermal Simulation Analysis

TMG/I-DEAS is a piece of thermal simulation software based on the Monte Carlo method (MCM) and the theory of radiative heat transfer [30,31], and was employed to analyse the solar heat flux on the satellite-borne lasercom sensor. The numerical equations are as follows [32]:

(4) $$\left\{\begin{array}{l} \frac{{\left[kA\frac{\partial T}{\partial x}\right]}_{x-\Delta x}-{\left[kA\frac{\partial T}{\partial x}\right]}_{x}}{\Delta x}+q_{rad}A=\rho cA\frac{\partial T}{\partial x}\\ \frac{{\left[kA\frac{\partial T}{\partial y}\right]}_{y-\Delta y}-{\left[kA\frac{\partial T}{\partial y}\right]}_{y}}{\Delta y}+q_{rad}A=\rho cA\frac{\partial T}{\partial y} \end{array}\right.,$$

In this expression, *T* represents the temperature, (*x*, *y*) represents the node on a geometric surface, *k* is the thermal conductivity coefficient, *ρ* is the density, *c* is the specific heat capacity, and *q_rad_* represents the radiative heat transfer energy density:

(5) $$q_{rad}=\sigma A\varepsilon F,$$

In this expression, *σ* is a Stefan Boltzmann constant with a value of 5.67 × 10^−8^ W/(m^2^·K^4^), *ε* is surface emissivity, and *F* represents the radiation heat transfer angular coefficient, which is the proportion of radiant energy emitted by surface *i* that is projected onto surface *j* and subsequently absorbed by surface *j*. This can be calculated using the following formula:

(6) $$F_{ij}=\frac{1}{A_{i}}\int_{A_{i}}\int_{A_{j}}\frac{cos\left(\theta_{i}\right)cos\left(\theta_{j}\right)}{\pi r^{2}}dA_{j}dA_{i},$$

In this expression, *A_i_* and *A_j_* refer to the areas of surfaces *i* and *j*, respectively, and *θ_i_* and *θ_j_* are the angles between the normal vectors of surface *i* and surface *j* and the distance between the two surfaces, respectively. From the definition of the radiation heat transfer angular coefficient and its calculation formula, it can be seen that this value is a function of the radiant heat transfer relationship from one surface, *i*, to another, *j*. Its key features are its relativity and completeness.

The radiation heat transfer angular coefficients were solved based on the MCM, and can thus be rewritten as follows:

(7) $$F=\frac{1}{N}\sum_{n=1}^{N}g_{D_{s}}({\left(x_{1},y_{1}\right)}^{\left(n\right)},{\left(x_{2},y_{2}\right)}^{\left(n\right)},\cdots ,{\left(x_{n},y_{n}\right)}^{\left(n\right)}),$$

In this expression, (*x_i_*,*y_i_*) is randomly distributed on the geometric plane of *D_s_*, with each point corresponding to a function value, g(*x_i_*,*y_i_*). Provided that a sufficiently large number of nodes (*x_i_*,*y_i_*) are taken, the averaging method can be employed to yield accurate evaluations of the radiation heat transfer angular coefficients. The probability and rate of convergence of this method are independent of the dimensionality of the problem. Furthermore, this numerical method is highly adaptable and stable in the integration of multidimensional complex surfaces.

In order to evaluate the effectiveness of the thermal control solution, extreme operating conditions were employed in the thermal simulation as the analysis conditions. According to the characterisations of the radiation heat flux presented in Section 3, the solar heat flux density is greatest during the vernal equinox, while the optical axis orientation (+Z direction) receives a significant amount of solar heat flux 24 h a day during the winter solstice. Therefore, these two extreme operating conditions were adopted in our thermal simulation analysis to evaluate the heat dissipation capability of the thermal control solutions, and the specific setup parameters of these two extreme operating conditions are presented in Table 4.

**Figure 12:**
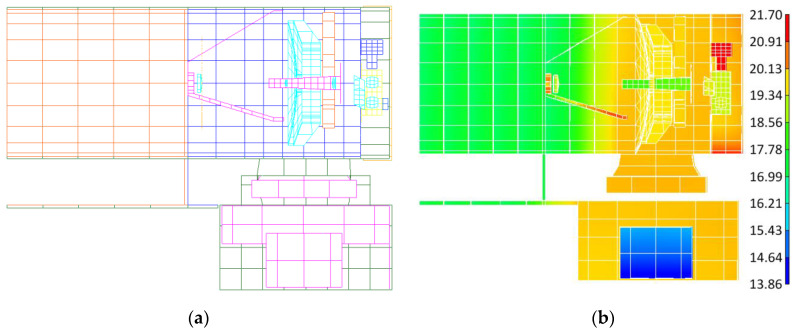
Finite element thermal simulation. (**a**) Finite element model and (**b**) temperature distribution contour of the lasercom sensor.

**Figure 13:**
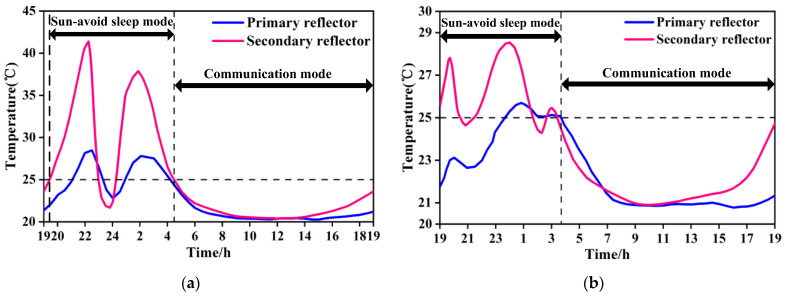
Temperature curve of the reflectors. (**a**) Temperature change in the reflectors during the spring equinox and (**b**) temperature change in the reflectors during the winter solstice.

A finite element model of the lasercom sensor for thermal simulation was created in TMG/I-DEAS (6.8 NX Series) software and the model was fully meshed with shell elements, as shown in Figure 12a. The simulation results are presented in Figure 12b and Figure 13. Figure 12b illustrates the temperature distribution contour of the lasercom sensor at a typical operating time (12:00 local time at the sub-satellite point) in communication mode, and Figure 13 shows the temperature curve of the reflectors in a 24 h cycle.

From Figure 13a, it can be concluded that during the equinox period, the temperatures of the primary and secondary reflectors in the period of 19:30 p.m.~4:30 a.m. exceed the permissible operating temperature range in Table 2. Consequently, the lasercom sensor is in the Sun-avoid sleep mode during this period, where the temperatures of the primary reflector and secondary reflectors are controlled within the ranges of 15~35 °C and 15~50 °C, respectively, which meets the temperature index requirements of the Sun-avoid sleep mode in Table 2. The temperatures of the primary and secondary reflectors are maintained within a stable range of 20 to 25 °C from 4:30 a.m. to 19:30 p.m., and the lasercom sensor is in the satellite–ground laser communication mode during this period. Therefore, the thermal permissible satellite–Earth optical communication hours per day is 15 h/d during the spring equinox period. From Figure 13b, it can be concluded that during the solstice period, the temperature of the primary and secondary reflectors is maintained within a stable range of 20 to 25 °C after 3:40 a.m., which meets the temperature index requirements of the communication mode in Table 1, and the thermal difference between the primary and secondary reflectors is less than 3.8 °C, so the communication mode period is from 3:40 a.m. to 19:00 p.m. Therefore, the thermal permissible lasercom duration is 15.3 h/d during the winter solstice period.

## 5. Direct Sunlight Avoidance Strategy

The solar heat flux characteristics, as illustrated in Figure 6 above, indicate that the optical axis orientation (+Z direction) is exposed to direct sunlight around midnight (20:00 p.m. to 4:00 a.m. local time at the sub-satellite point), with maximum solar heat flux. Direct sunlight striking the primary reflector has the potential to damage the film of the reflector, resulting in the lasercom sensor becoming inoperative or even permanently disabled. Thus, it is necessary to rotate the antenna’s optical axis orientation to avoid direct sunlight.

### 5.1. Direct Sunlight Avoidance Strategy during the Equinox

The solar heat flux characteristics, as shown in Figure 6 above, indicate that not only will the phenomenon of direct sunlight not occur at midnight during the equinox, but the ‘midnight Sun disappearance’ phenomenon will take place, at which time the Sun, Earth, and satellite will be in alignment, resulting in the sensor being shielded from solar radiation and heat flux, which will enable satellite–ground communication. Nevertheless, there is still a considerable amount of near-direct sunlight entering the optical antenna for approximately four hours around midnight, when a solar avoidance strategy is required.

**Figure 14:**
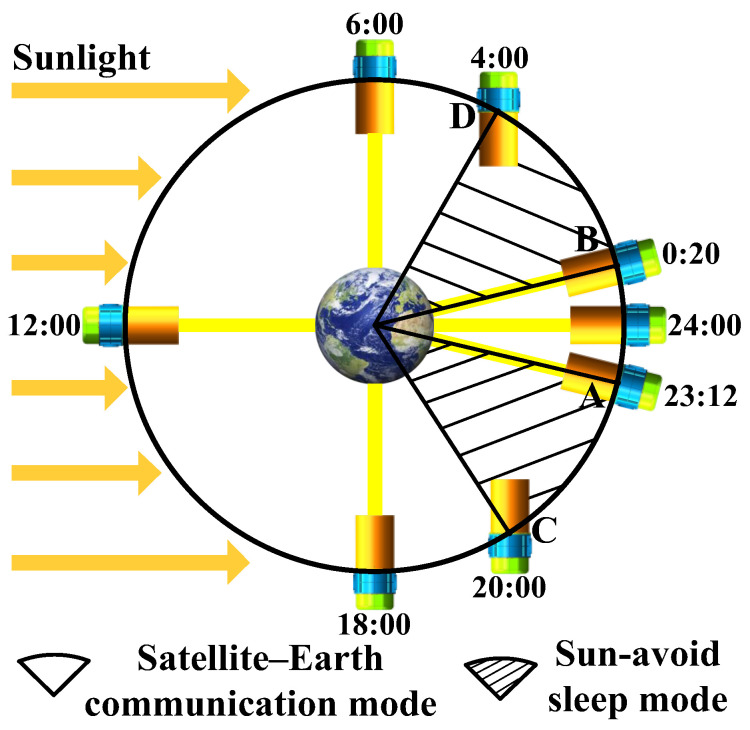
The solar avoidance strategy.

**Figure 15:**
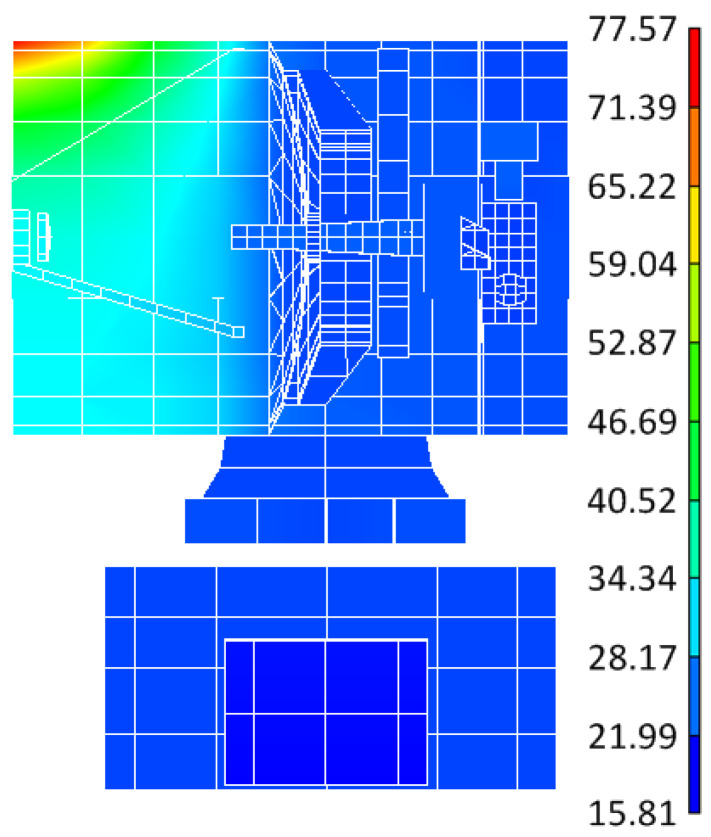
Temperature contour of C/D point.

The solar avoidance strategy during the equinox is shown in Figure 14, with $\overset{◠}{\mathrm{AB}}$ representing the ‘midnight Sun disappearance’ period, during which satellite–ground communications are possible. The shaded area in Figure 14 represents the Sun avoidance period, during which the lasercom sensor must rotate the antenna’s optical axis orientation and enter Sun-avoid sleep mode. The M/N point represents the moment of entering/leaving the Sun avoidance period, during which the solar incidence angle is much less than the ‘solar-blocking angle’, with the baffle unable to block sunlight. Figure 15 illustrates the temperature distribution contour of the C/D point.

Figure 13 illustrates that the period from 4:30 a.m. to 19:30 p.m. is the satellite–ground communication period and the thermal permissible lasercom duration is 15 h/d. However, by adopting the direct sunlight avoidance strategy, namely, initiating communication at 23:12 p.m. and terminating it at 00:20 a.m., the communication time can be extended by nearly 70 min. Therefore, the thermal permissible lasercom duration can be extended to 16 h/d during the equinox period.

### 5.2. Direct Sunlight Avoidance Strategy during the Solstice

**Figure 16:**
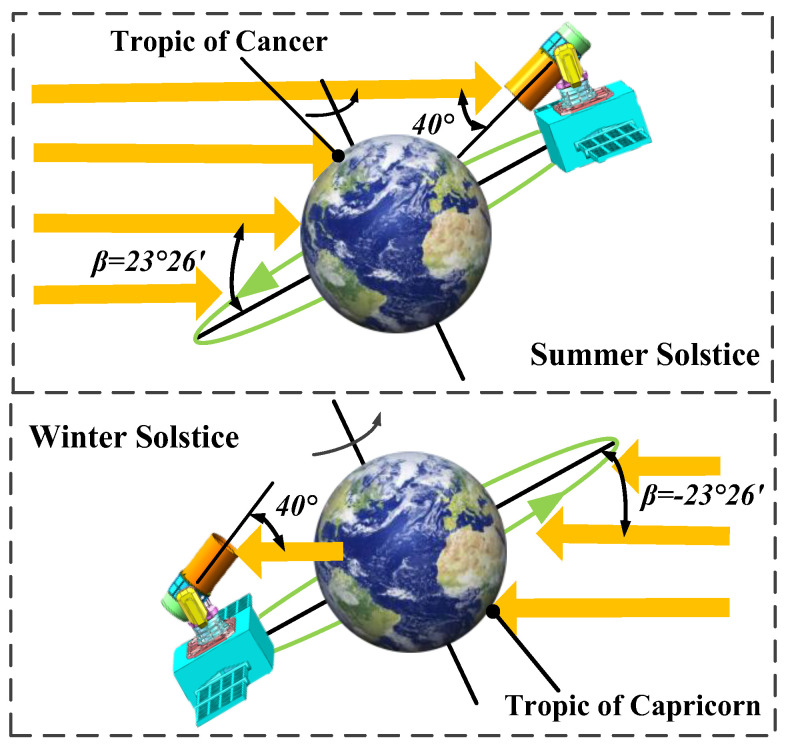
The solar avoidance strategy employed around midnight during the solstice.

**Figure 17:**
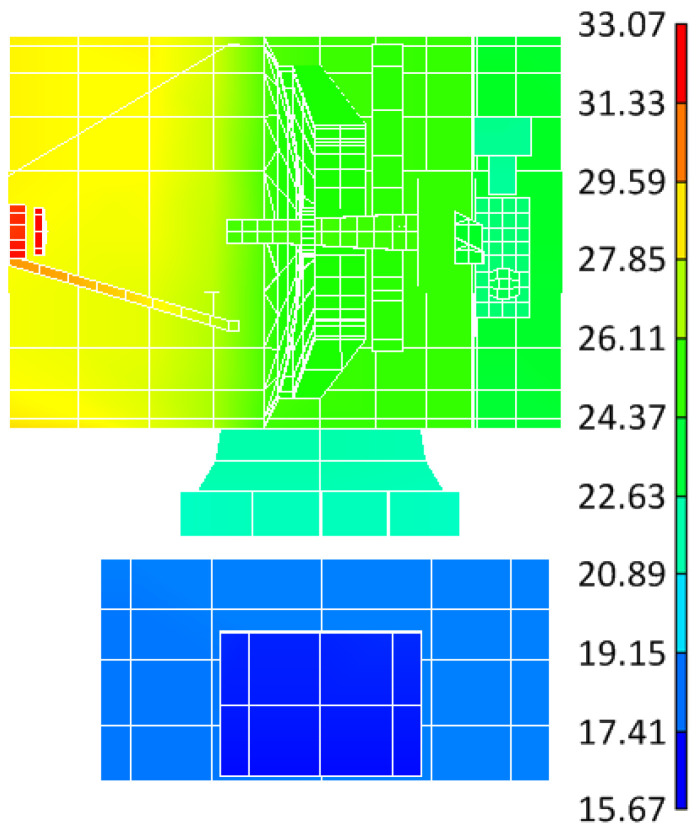
Temperature contour of the lasercom sensor during the solar avoidance period.

According to the characterisation of the space radiation heat flux presented in Section 3, if *β* ≥ |±8.8°|, there will be no ‘midnight Sun disappearance’. Here, *β* = 23°26′ during the solstice, so it is obvious that there is no ‘midnight Sun disappearance’ phenomenon; thus, the solar avoidance strategy must be employed around midnight. This is shown in Figure 16. During the summer solstice, the sun shines in the northern hemisphere, thus requiring the antenna’s optical axis orientation to be deflected by 40° to the south. Conversely, during the winter solstice, the Sun shines in the southern hemisphere, necessitating the antenna’s optical axis orientation to be deflected by 40° to the north. The temperature distribution contour of the lasercom sensor during the solar avoidance period is shown in Figure 17.

From Figure 17, it can be concluded that during the solar solstice avoidance period, the temperature of the primary and secondary reflectors exceeds the permissible operating temperature range in Table 2. Consequently, the lasercom sensor is in the Sun-avoid sleep mode during this period and meets the temperature index requirements of the Sun-avoid sleep mode in Table 1.

## 6. Conclusions

A combined passive and active thermal control solution for a GEO satellite-borne lasercom sensor based on the sensor’s orbital parameters and performance requirements was presented in this paper, and its temperature distribution contour under extreme operating conditions was simulated using IDEAS-TMG. The simulation results demonstrated that the temperatures of the primary and secondary reflectors of the lasercom sensor in communication mode can be stably controlled within the permissible operating temperature range. This paper also presented a strategy for avoiding direct sunlight around midnight, under which the temperatures of the primary and secondary reflectors are controlled within the temperature index requirements in Sun-avoid sleep mode. Therefore, the thermal control solution and solar avoidance strategy proposed in this paper achieved long-term fine-stable control of the temperature field of the optical antenna of the GEO satellite-borne lasercom sensor, which met the thermal permissible hours per daily orbit cycle for communication in excess of 14 h/d.

## Figures and Tables

**Table 1:** Optical antenna material properties table.

Material	Components	Density (×10^−6^ kg/mm^3^)	Specific Stiffness (kN·m/g)	Thermal Conductivity (W/m·°C)	Expansivity (×10^−6^/°C)	Specific Heat Capacity (J/Kg·°C)
Silicon carbide	Primary/secondary reflector	3.05	131.2	185	2.5	821
Indium steel	Reflector mount	8.1	25.6	13.9	2.4	0
Titanium alloy	Reflector cell, three-vane spider	4.44	25.9	5.4	7.9	611
Carbon fibre	Baffle, radiating plate	1.6	89	35	0.1	500
Polyimide	Insulating spacer	1.35	2.3	0.32	38.6	/

**Table 2:** Optical antenna parameters and performance requirements.

**Relevant parameters**				
**Component**		**Parameters**	**Component**	**Parameters**
Wavelength (beacon-tracking unit)		800 nm	Aperture	250 mm
Wavelength (communication unit)		1500 nm	Acquisition field of view	4 mrad
Lasercom sensor mount surface temperature				−10 °C~10 °C
**Performance requirements**				
**Mode**	**Primary reflector**		**Secondary reflector**	
Communication mode	22.5 ± 2.5 °C		22.5 ± 2.5 °C	
	Temperature uniformity ≤ 5 °C		Temperature difference from primary mirror ≤ 5 °C	
Sun-avoid sleep mode	15–35 °C		15–50 °C	
Optical antenna thermal control power				≤50 W
**Thermal permissible laser communication hours per daily orbit cycle**				≥14 h/d

**Table 3:** Active thermal control power consumption table for the optical antenna.

Serial Number	Temperature Control Area	Power Consumption (W)
1	Baffle	10
2	Primary reflector cell	8
3	Primary reflector mount	4
4	Secondary reflector baffle	2
5	Secondary reflector mount	2
6	ATP subsystem	4
7	Total	30

**Table 4:** Operating conditions’ parameters for the thermal simulation analysis.

Case Name	Operating Conditions’ Parameters	Characterisation
Vernal equinox	(1) Solar heat flux density = 1367 W/m^2^ (2) The angle (*β*) between the sunlight and the Earth’s equatorial plane = 5° (3) Yellow film is wrapped over the outermost layer of the MLI, and its solar absorptivity (*α_s_*) is 0.36 (4) KS-Z white paint is coated over the heat-expelling surface of the radiating plate, and its solar absorptivity (*α_s_*) is 0.13	(1) Sunlight shines directly over the Earth’s equator with the maximum solar heat flux (2) The ‘midnight Sun disappearance’ phenomenon occurs for up to 72 min.
Winter Solstice	(1) Solar heat flux density = 1322 W/m^2^ (2) The angle (*β*) between the sunlight and the Earth’s equatorial plane = −23.43° (3) Yellow film is wrapped over the outermost layer of the MLI, and its solar absorptivity (*α_s_*) is 0.36 (4) KS-Z white paint is coated over the heat-expelling surface of the radiating plate, and its solar absorptivity (*α_s_*) is 0.13	(1) There is no ‘midnight Sun disappearance’ and the sensor is not in the shadow zone (2) Laser sensor exposed to solar heat flux 24 h a day

## Data Availability

The data may be provided free of charge to interested readers by request to the corresponding author’s email.
